# The Secondary Structure of a Major Wine Protein is Modified upon Interaction with Polyphenols

**DOI:** 10.3390/molecules25071646

**Published:** 2020-04-03

**Authors:** Mattia Di Gaspero, Paolo Ruzza, Rohanah Hussain, Claudia Honisch, Barbara Biondi, Giuliano Siligardi, Matteo Marangon, Andrea Curioni, Simone Vincenzi

**Affiliations:** 1 Department of Land, Environment, Agriculture and Forestry (TESAF), University of Padua, Viale dell’Università, 16, 35020 Legnaro (PD), Italy; mdgdigaspero@gmail.com; 2 Institute of Biomolecular Chemistry of CNR, Padua Unit, via Marzolo 1, 35131 Padua, Italy; paolo.ruzza@unipd.it (P.R.); claudia.honisch@phd.unipd.it (C.H.); barbara.biondi@unipd.it (B.B.); 3 Diamond Light Source Ltd., Harwell Science and Innovation Campus, Didcot, Oxfordshire OX11 0DE, UK; rohanah.hussain@diamond.ac.uk (R.H.); giuliano.siligardi@diamond.ac.uk (G.S.); 4 Department of Chemical Sciences, University of Padua, Via Marzolo 1, 35131 Padua, Italy; 5 Department of Agronomy, Food, Natural Resources Animals and Environment (DAFNAE), University of Padua, Viale dell’Università, 16, 35020 Legnaro (PD), Italy; andrea.curioni@unipd.it (A.C.); simone.vincenzi@unipd.it (S.V.)

**Keywords:** polyphenols, VVTL1, Synchrotron Radiation Circular Dichroism (SRCD), protein interaction, protein structure, wine

## Abstract

Polyphenols are an important constituent of wines and they are largely studied due to their antioxidant properties and for their effects on wine quality and stability, which is also related to their capacity to bind to proteins. The effects of some selected polyphenols, including procyanidins B1 and B2, tannic acid, quercetin, and rutin, as well as those of a total white wine procyanidin extract on the conformational properties of the major wine protein VVTL1 (*Vitis vinifera* Thaumatin-Like-1) were investigated by Synchrotron Radiation Circular Dichroism (SRCD). Results showed that VVTL1 interacts with polyphenols as demonstrated by the changes in the secondary (far-UV) and tertiary (near-UV) structures, which were differently affected by different polyphenols. Additionally, polyphenols modified the two melting temperatures (T_M_) that were found for VVTL1 (32.2 °C and 53.9 °C for the protein alone). The circular dichroism (CD) spectra in the near-UV region revealed an involvement of the aromatic side-chains of the protein in the interaction with phenolics. The data demonstrate the existence of an interaction between polyphenols and VVTL1, which results in modification of its thermal and UV denaturation pattern. This information can be useful in understanding the behavior of wine proteins in presence of polyphenols, thus giving new insights on the phenomena that are involved in wine stability.

## 1. Introduction

Polyphenols are important secondary metabolites of higher plants that are extensively studied for their antioxidant properties and related health benefits [1]. In grapes, polyphenols are generally present in skins and seeds, from where they are extracted at different extents during winemaking, significantly contributing to the physical-chemical and organoleptic properties of resulting wines [2,3]. In addition to their role in determining wine color, the capability of polyphenols to bind to proteins greatly affects wine astringency [4,5], and it is exploited for wine fining with proteinaceous agents [6], an operation that consists in adding proteins (e.g., gelatin, casein, albumin) to a wine to remove phenolic molecules (e.g., flavan-3-ol derivatives and condensed tannins), thus improving its organoleptic and stability properties [4,7,8]. Moreover, the binding of polyphenols to the grape-derived proteins that are present in wines has been demonstrated [9,10], even if the effects of this occurrence are not fully understood. For example, the low amounts of polyphenols that are found in white wines (200–500 mg/L), in particular polymers of (+)-catechin and (−)-epicatechin [11], have been associated to protein haze formation, a major problem that affects white wines’ quality. This phenomenon starts with a change in the structure (unfolding) of the wine proteins, but the possible role of polyphenols during this step is not clear [12,13]. The current model of protein haze formation suggests that the exposition of hydrophobic sites on the unfolded proteins makes them susceptible to aggregation. These aggregates gradually grow through the cross-linking caused by interactions with other wine components, possibly including polyphenols [12,14,15]. In contrast, the phenomenon of haze formation is not considered to be a problem for red wines, which, despite containing similar amounts of proteins when compared to white wines [16], seem to be stable with regard to protein precipitation [17]. The reason for this difference is still largely unknown, but it can be hypothetically related to the higher quantity of those components, mainly phenolics and polysaccharides, released from skin and seeds during red wine maceration.

**Figure 1:**
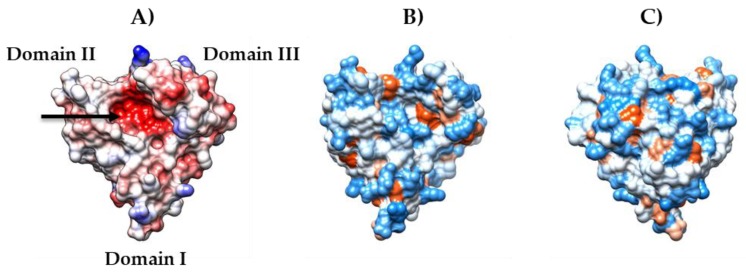
(**A**) Mapping of the electrostatic potentials on the molecular surface of the grape VVTL1 4L5H. The negative and positive potentials are colored in red and blue, respectively, while neutral areas are in white. The arrow indicates the acidic cleft located between domains I and II. **(B**, **C)** Mapping of the surface hydrophobicity of the VVTL1 4L5H: (**B**) front view; and, (**C**) back view (rotated approximately 180˚). Hydrophobicity continuum starts from dodger blue for the most hydrophilic areas, to white, to orange and to red for the most hydrophobic areas. The images were generated using the Chimera 1.14 software [27].

Most of the proteins that are found in wines are grape pathogenesis-related (PR)-proteins, mainly Thaumatin Like Proteins (TLPs) and chitinases [14,18,19]. These proteins withstand the vinification process and persist in the wine after bottling [12,20,21]. The most abundant proteins generally found in wines belong to the TLP family, a class of proteins with molecular mass of about 21 kDa. In particular, *Vitis vinifera* Thaumatin Like Protein 1 (VVTL1) seems to be the major component of most wines, either white [22] or red [23]. At least in white wines, different isoforms of wine VVTL1 have been shown to possess different heat stability, with the isoform 4JRU being more unstable (T_M_ 56 °C) than isoforms 4L5H and 4MBT (T_M_ 62 °C) [24,25]. VVTL1 isoforms all have three structural domains (a central core flanked by two domains, Figure 1A) and eight disulphide bridges, which give stability to the structure [24]. These proteins present surface areas that are characterized by different hydrophobicity (Figure 1B,C) and electrostatic potentials (Figure 1A), and show an acidic cleft (see arrow in Figure 1A), in which small molecules, such as phenolics, can be accommodated [26].

The interactions between proteins and phenolics have been mainly studied considering polymeric flavanols as tannins. These interactions can be either reversible or irreversible and they do not necessarily lead to protein precipitation. A comprehensive review on the interactions between polyphenols and macromolecules has been published by Le Bourvellec & Renard [28]. In the case of reversible interactions, non-covalent forces, such as hydrogen bonding, hydrophobic interactions, and van der Waals forces, are involved. NMR studies indicated the presence of stacking interactions of the phenolic rings with the proline residues and the stabilization of the complexes through hydrogen bonding between the H-acceptor site of the adjacent peptide bond and the hydrogen atom of the phenolic hydroxyl [29]. Isothermal titration calorimetry studies showed that the interaction of flavanols with poly-l-proline involves both entropic (associated to the hydrophobic effect and conformational changes, mainly involving flavanols polymers) and enthalpic (attributed to hydrogen bonding, mainly involving flavanols monomers) phenomena [30]. In the case of irreversible interactions, a covalent bond is instead formed between the proteins and products of polyphenols oxidation [31].

In general, proteins that are involved in the interaction with polyphenols have been shown to be proline-rich and/or quite hydrophobic, with a conformationally open and flexible structure [31,32]. However, as previously mentioned, the wine proteins do not fully possess these features, and so they deserve to be investigated, as they cannot be included in the general studies previously mentioned [31,32].

**Figure 2:**
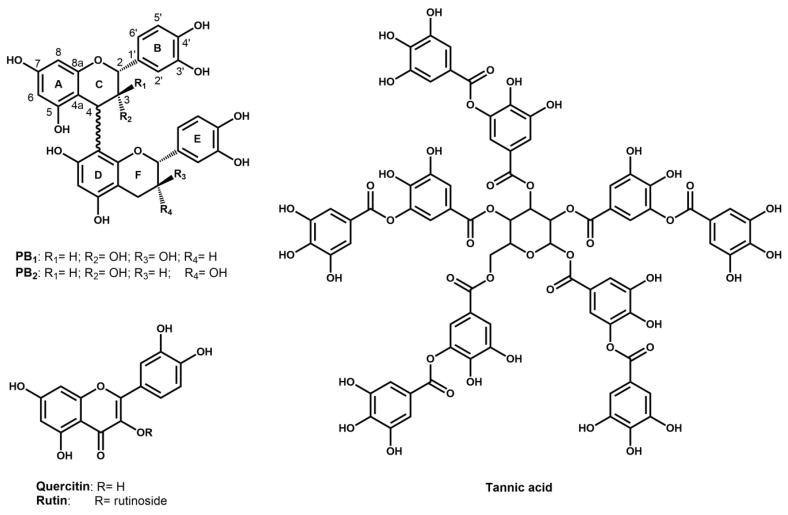
Chemical structures of proanthocyanidins B1 (PB1) and B2 (PB2), quercetin, rutin, and tannic acid.

Proanthocyanidins B1 and B2 are flavan-3-ols (catechin or epicatechin) dimers that are present in grapes and wines in varying amounts [33]. These compounds are formed by two units condensed by a C4–C8 linkage (Figure 2) [34]. In particular, proanthocyanidins B2 are composed by two units of (−)-epicatechin, whereas proanthocyanidins B1 differ by the presence of (+)-catechin in the terminal unit of the chain [34]. NMR and molecular modelling studies showed that these molecules might exist either in compact or extended conformation. The compact form is characterized by the presence of two NOE correlation peaks between proton 2C and protons 2′E and 6′E, correlation that disappears in the extended form [35]. The amount of each conformation is strictly related to the medium composition. In hydro-alcoholic solutions, such as wine, proanthocyanidins B1 adopts a dominant compact conformation (92%), whereas proanthocyanidins B2 manifests both extended and compact form (45:55%) [36].

Tannic acid belongs to the family of hydrolysable tannins, molecules that are composed of phenolic acids, as gallic acid, which are esterified to polyols (typically glucose) (Figure 2). Hydrolysable tannins are not naturally present in wine, but they can be added during winemaking via direct addition and/or via extraction from wood during ageing in barrels.

Quercetin and rutin are flavonols that are characterized by the 3-hydroxyflavan backbone (Figure 1). Flavonols in grapes exist as 3-glycosides, whereas the corresponding free aglycones can be found in wines, along with the 3-glycosides, as a result of acid hydrolysis that occurs during winemaking and aging [37].

This study investigates the interactions between some wine polyphenols, including those above mentioned, and the main wine protein VVTL1 by Synchrotron Radiation Circular Dichroism (SRCD), in order to increase the knowledge on how polyphenols interact with wine proteins.

## 2. Results and Discussion

### 2.1. Polyphenols-VVTL1 Interactions

CD spectroscopy is a well-known technique for studying protein folding and secondary structure. Moreover, its high sensitivity to sample perturbation allows for investigating protein structures as a function of temperature, solvent composition, chemical agents and ligand interactions [38]. The high photon flux and brilliance of the synchrotron radiation used in SRCD spectroscopy allows for obtaining a high signal to noise ratio when compared to bench-top instruments. Additionally, a photo-denaturation of the ordered proteins is observed when consecutive repeated scans are collected, providing useful information on the protein photo-stability as well as on the influence of ligands and medium composition [39]. These techniques have been adopted here for the investigation of the interactions of a major wine protein (VVTL1) with a total wine tannin extract (WTE) and five individual phenolic compounds that were chosen based on their structural diversity.

It was possible to calculate two important parameters for the interpretation of the behavior of this protein based on the sequence and structural information available for the VVTL1 isoform used in this study [40]. In particular, the GRAVY (Grand Average of hydropathy) value was found to be -0.438, and the instability index was 34.31. These values indicate that VVTL1 is a hydrophilic stable protein [41,42]. Moreover, this protein is characterized by a relative rigid structure due to the presence of the eight disulphide bonds [24] that, alongside with its hydrophilicity, should not favor its interaction with polyphenols, even if it has been reported that the protein possesses a pocket with high hydrophobicity that could host/bind small polyphenols as quercetin and caffeic acid [26].

The far-UV SRCD spectrum of VVTL1 in model wine solution (MWS) has been described in our previous work [43]. This spectrum showed two positive bands at about 195 nm and 231 nm, and a negative band at 213 nm. The bands at 195 and 213 nm, which are attributable to the amide bond, are characteristic of a β-sheet conformation, while the positive band at 231 nm is due to the contribution of aromatic side-chains [44].

**Figure 3:**
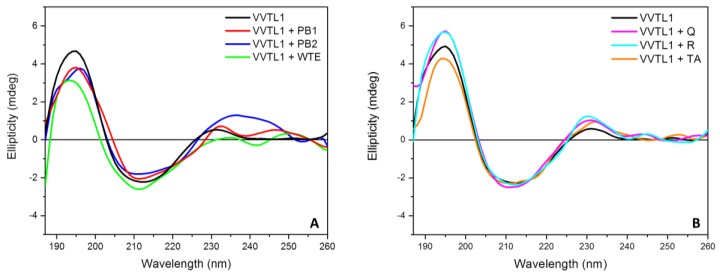
Far-UV Synchrotron Radiation Circular Dichroism (SRCD) spectra of VVTL1 (0.400 mg/mL) alone (black line in both pictures) or in presence of 2 eq. of polyphenols (**A**: PB1 in red, PB2 in blue, wine total extract (WTE) in green; **B**: Q in magenta, R in cyan, TA in orange) in model wine solution (MWS). Spectra were recorded at 20 °C using a Suprasil 0.02 cm cell (Hellma) filled with 60 µL of solution, integration time 1 s, 1 nm digital resolution, 39 nm/min. scan speed, and monochromator slit widths 1.2 nm bandwidth.

In this work, VVTL1 was tested by far-UV SRCD in the presence of single different polyphenols as well as in the presence of a total tannins wine extract (WTE). After the subtraction of the signals produced by the individual polyphenols, the spectra shown in Figure 3 were obtained.

Little differences were detected in the presence of polyphenols, being visible mainly in the positive band at 195 nm. In particular, the presence of PB1, PB2, WTE, and tannic acid (TA) caused a decrease in the intensity of the 195 nm band, while both quercetin (Q) and rutin (R) had the opposite effect. In contrast, major changes in the negative band at 213 were never detected (Figure 3). The same experiments were also performed at 5 °C and 70 °C (see Section 2.2).

The SRCD spectral data were used to calculate variations in the protein secondary structure content deriving from the presence of the different polyphenols that were tested at different temperatures (Table 1).

The data of Table 1 indicate that the presence of polyphenols determines a small decrease in the β-sheet content of VVTL1 as the temperature increased, whereas more important variations were visible for the unordered structure at 70 °C for all of the phenolic compounds tested. It is likely that this occurrence is due to the biding of these phenolics to the heat-unfolded VVTL1. Indeed, there seems to be a cooperative effect between the temperature and presence of certain phenolics (PB1, PB2, WTE, and TA), which results in an increase of the unordered structure.

**Figure 4:**
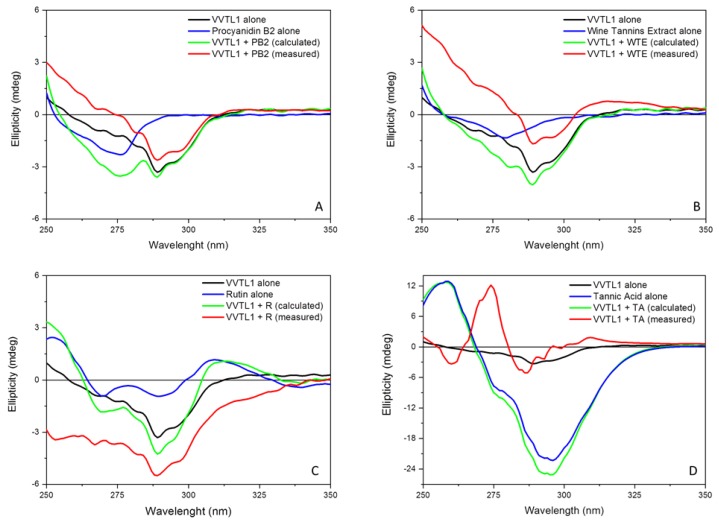
Near-UV CD spectra in MWS of polyphenols alone (blue lines), VVTL1 alone (black lines) and VVTL1 in presence of 2 eq. of selected polyphenols, measured (red lines) or calculated (green lines). Polyphenols were Procyanidin B2 (PB2) (**A**), Wine Tannins Extract (WTE) (**B**), Rutin (R) (**C**), and tannic acid (TA) (**D**). PB1 and Quercetin are not shown as the differences between calculated and measured spectra were equivalent to those shown in (**A**) and (**B**), respectively. VVTL1 concentration was 0.500 mg/mL. The CD spectra were measured using nitrogen flushed Jasco J-715 spectropolarimeter, scanning speed 50 nm/min., data pitch 0.5 nm, response time 4 s, bandwidth 2 nm using a Suprasil 1.0 cm cell (Hellma) that was filled with 900 µL of solution.

While the far-UV CD spectra were not strongly affected by the addition of polyphenols, this was not the case for the near-UV (230–250 nm) CD spectrum of VVTL1 (Figure 4). This region, which shows the contribution of the side-chains of the aromatic residues [44], was strongly modified after the addition of polyphenols, which suggests that the interaction between VVTL1 and polyphenols modified the aromatic environment. In particular, the data showed that a negative band at 290 nm, with a shoulder at about 300 nm, characterized VVTL1, while, below 260 nm, it exhibited a positive ellipticity. In the presence of either procyanidins PB1 or PB2, the measure of the near-UV CD spectrum of VVTL1 (corrected for the signal of PB1 and PB2 alone) showed a decrease in the intensity of both the negative band and shoulder, while the positive signal was increased and shifted at higher wavelengths (Figure 4). This confirms the involvement of aromatic residues on the protein interaction with polyphenols already seen in the far-UV spectra. This conclusion results from the comparison between the calculated CD spectrum (as the sum of the individual VVTL1 and polyphenols CD spectra), and the experimentally measured CD spectrum of the VVTL1-polyphenol mixture. Usually, if two analytes do not interact with each other in solution, the spectrum of a solution containing both analytes is equal to the algebraic sum of the individual contributions. This was not the case in this study, as, for every polyphenol tested, the calculated and the measured spectra differed, thus indicating the interaction between the analytes that are present in solution. This difference confirms that an interaction occurs, and indicates a change in the aromatic residues environment.

For both total WTE (Figure 4B, red line) and quercetin (Q data not shown), similar shapes for the curves (calculated and measured) were detected, while in presence of the quercetin glycoside rutin (R), the measured near-UV CD spectrum of VVTL1 was profoundly modified when compared to the calculated one (Figure 4C, red and green lines). In particular, the intensities of both the negative band at 290 and the shoulder at 300 nm were greatly increased, and two non-resolved negative bands at about 255 and 265 nm appeared. The different behavior of quercetin and rutin might be attributable to the presence of the glycoside moiety in rutin that could increase hydrogen bonding with the VVTL1 protein. Additionally, in the presence of TA the near-UV CD spectrum of VVTL1 also resulted strongly modified (Figure 4D, red line), being characterized by two positive bands at about 275 and 309 nm and three negative bands at 261, 287, and 293 nm. This different spectrum should be attributed to the peculiar structure of tannic acid, a compound that belongs to the so-called hydrolysable tannins extracted from oak, which are chemically different from the other grape-derived polyphenols used in this study.

Taken together, these data confirm that VVTL1 interacts with polyphenols, as demonstrated by the changes in the secondary (far-UV) and tertiary (near-UV) structures of the protein, and show that different polyphenols induce different changes on both of these structures. This indicates that the chemical structure of individual phenolic compounds is responsible for different interactions with VVTL1, which should occur on different regions of the protein structure.

### 2.2. Thermal and UV-Denaturation Assays

The denaturation assays were conducted to assess whether the stability of the VVTL1 protein is affected by its interaction with the different polyphenols that were considered in this study. This information is of particular relevance, as VVTL1 stability is related to haze formation in wines [47], but the role of polyphenols in this mechanism has not yet been fully elucidated [12,13]. Thermal and UV denaturation experiments were carried out to clarify this point.

The buffer (MWS) and the range of temperatures (5–70 °C) for the thermal stability studies were selected taking into account the physico-chemical conditions existing in wine as well as the possible temperatures reached during wine storage and transportation [25,48].

**Figure 5:**
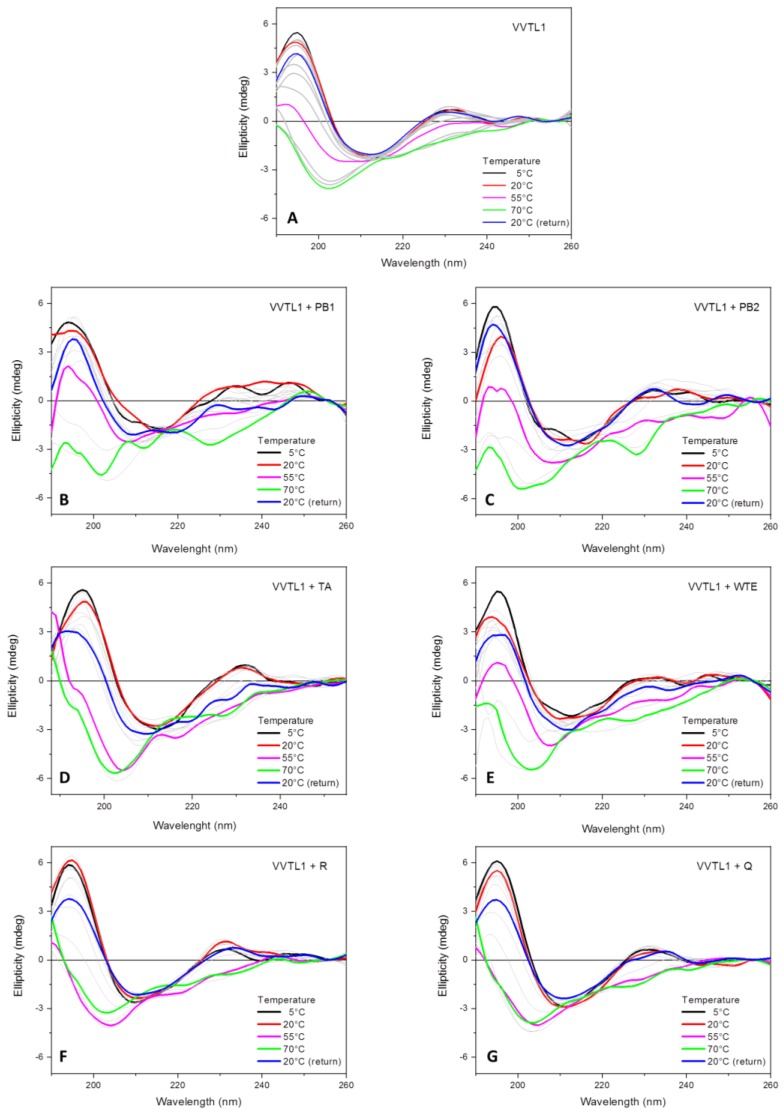
Thermal denaturation experiments: far-UV SRCD spectra of VVTL1 (0.400 mg/mL in MWS) alone (**A**) and in presence of Procyanidin B1 (PB1) (**B**) and B2 (PB2) (**C**), tannic acid (TA) (**D**), Wine Tannins Extract (WTE) (**E**), Rutin (R) (**F**), and Quercetin (Q) (**G**) were recorded at different temperatures (indicated) using a Suprasil 0.02 cm cell (Hellma) filled with 60 µL of solution, integration time 1 s, 1 nm digital resolution, 39 nm/min scan speed and monochromator slit widths to 1.2 nm bandwidth. Grey lines indicate intermediate temperatures with steps of 5 °C.

A drastic change in the far-UV SRCD spectrum of VVTL1 alone is detectable at a temperature starting from 60 °C. Above this temperature, the two positive bands at 195 and 231 nm disappeared, the negative band at 213 nm was shifted to a lower wavelength (about 202 nm) and a shoulder appeared at about 218 nm (Figure 5A). This drastic change of VVTL1 corresponds to an equally drastic reduction in the ordered secondary structure (β-sheets) of the protein (see Table 1 and Supplementary Materials Figure S1). This is coherent with the melting temperature of VVTL1 [25].

The addition of different phenolic compounds resulted in a modification of the response of VVTL1 to thermal denaturation, as measured by its far-UV SRCD spectra (Figure 5). This indicates that phenolics interact with the protein, thus modifying its conformation and stability. In particular, some phenolic compounds increased the VVTL1 stability (PB1, PB2, and WTE), while others decreased it (TA, Q, and R), as visible by the T_M_ values (Table 2).

The SRCD spectra of VVTL1 at 20 °C that were recorded before and after the 70 °C denaturation assay were almost superimposable (Figure 5A), thus suggesting a partial reversibility for the protein structure upon thermal denaturation. These findings are in strong agreement with previous studies on the thermal stability of VVTL1, in which it was highlighted that repeated DSC scans of a VVTL1 showed a high degree of reversibility of unfolding with the protein returning to native or near-native conformation [25].

Even the reversibility of the thermal denaturation process of VVTL1 was influenced by the addition of polyphenols. Indeed, in the presence of PB1, PB2, and WTE, the CD spectra recorded at 20 °C after heating at 70 °C did not significantly differ from the one recorded at the same temperature during the thermal analysis. This suggests that these polyphenols do not prevent the possibility for the protein to return to its native conformation upon cooling. On the contrary, TA, Q, and R seem to have a negative effect on the reversibility of the protein unfolding (Figure 5).

The different impact of polyphenols on the thermal stability of VVTL1 can be better appreciated by comparing the melting curves of VVTL1 in the presence or not of polyphenols (Table 2 and Figure S2).

The melting curve of VVTL1 alone can be fitted by the double Boltzmann equation indicating the presence of two equilibria with T_M_ values of 32.2 °C and 53.9 °C, respectively (Table 2 and Figure S2). This suggests the presence in VVTL1 of two domains with different heat sensitivity, a fact that should be taken into account when the relationship between protein thermal denaturation and haze formation in wines is discussed.

When looking at the first T_M_ equilibrium, which was 32.2 °C for VVTL1 alone (Table 2), only the addition of PB1 resulted in an increase in the T_M_ value (i.e., protein stability at these temperatures), while the other five phenolic compounds all negatively influenced this parameter. The second VVTL1 T_M_ value (Table 2) was instead increased in the presence of some polyphenols (PB1, PB2, and WTE), while others (TA, Q, and R) had the opposite effect. A hypothesis could be that different domains of the VVTL1 interact differently with the phenolic compounds.

Of particular interest, also for practical winemaking, is the behavior that is shown in the presence of the pool of white wine procyanidins (WTE), which strongly affected the T_M_ equilibria. In particular, the first T_M_ was the lowest among those tested, while the second the highest. These results suggest that the mixture of procyanidins as they are present in wine have the ability to modulate the stability of the protein by affecting its melting temperatures.

It is also important to note, for practical winemaking, that quercetin has been associated to the formation of precipitates in red wines, a negative occurrence that decreases their marketability. Quercetin can cause the formation of insoluble colloidal particles by interacting with hydrophobic proteins [49], and, in wines, quercetin precipitates have also been shown to contain amino acids [50]. Therefore, it can be hypothesized that the destabilization of the proteins by quercetin shown here could be involved in the formation of this wine precipitate.

Taken together, these findings indicate that the same protein (VVTL1) can be more or less unstable depending on the phenolic composition of a wine, a piece of information that could contribute in explaining why different wines with similar protein composition and concentration can have a different protein instability potential.

An additional investigation on the stability of VVTL1 was carried out by studying the photo-denaturation of the protein alone or in the presence of polyphenols. In this technique, the high UV photon flux produced by the beamline induces a photo-denaturation of ordered proteins when consecutive repeated scans are collected [51,52]. This is principally due to the free radical damage of the photosensitive tryptophan and tyrosine residues, the oxidation of amino acid residue side chains, as well as the cleavage of disulphide bridges [53,54]. Like thermal denaturation, photo-denaturation provides useful and complementary information on protein stability as well as on the effects of ligands and medium composition [55,56].

Figure S3 reports the SRCD UV-denaturation experiments on VVTL1 alone or in the presence of polyphenols. The variation of the dichroic signal at 195 nm as a function of the number of scans to better evaluate the effect of polyphenols on the photo-denaturation of VVTL1 is reported in Figure S4. The rate of VVTL1 photo-denaturation is significantly different in the presence of polyphenols as compared to that determined for the VVTL1 alone. This confirms the interaction between VVTL1 and polyphenols already highlighted in the thermal denaturation studies. In particular, the presence of PB1, PB2 and WTE decreased the slope of the fitted curve (Figure S4), suggesting a protective effect of these procyanidins towards the photo-denaturation of the VVTL1 protein, results that are in agreement with those presented in Table 2.

The effect of photo-denaturation on the secondary structure content of VVTL1 alone was compared with that determined in the presence of the different polyphenols. A significant decrease in the ordered conformation of VVTL1 alone (Table 3) has been detected after twenty scans, a result in line with those obtained via thermal denaturation (see Table 1). A decrease in the ordered conformation was also observed in the presence of polyphenols. In particular, with WTE, a large decrease in the ordered conformation of VVTL1 was detected after twenty consecutive scans. Surprisingly, the secondary structure of VVTL1 was only marginally affected in the presence of both rutin and quercetin (Table 3), which suggested a protective role of these polyphenols towards the denaturation effects of the synchrotron radiation. The stabilizing effect of polyphenols is larger when the VVTL1 is photo-denatured than when it is thermally-denatured. This can be due to a direct action of polyphenols on VVTL1 during photo-denaturation, resulting in the stabilization of its secondary structure, although an indirect effect that involves the quenching of the reactive species [57] produced by the synchrotron radiation [58] cannot be excluded.

## 3. Materials and Methods

### 3.1. Polyphenols and Protein Preparation

Procyanidins B1 and B2 were purchased from Extrasynthese (Genay, France), tannic acid, quercetin, and rutin were purchased from Sigma–Aldrich (Milan, Italy). The wine total extract (WTE) included the total wine procyanidins (polymeric catechins and epicathechins) that were purified starting from 1.5 L of a Sauvignon blanc wine provided by a commercial winery in the Conegliano area (Italy). The wine was passed on a glass column (400 × 24 mm) packed with 20 g of Sephadex LH-20 resin (Sigma-Aldrich, Milan, Italy) previously equilibrated with 20% ethanol in water (*v*/*v*). The column was then washed with 2 column volumes (CV) of 20% ethanol (*v*/*v*) and 2 CV of absolute ethanol before eluting the total wine procyanidins with 2 CV of 60% acetone in water (*v*/*v*). Acetone was then evaporated from the procyanidins solution (at 40 °C by Büchi Rotavapor R-114, Flawil, Switzerland), and the resulting solution was frozen and then freeze dried.

VVTL1 (PDB: 4L5H) was purified from a Manzoni Bianco wine provided by a commercial winery in the Conegliano area (Italy) and characterized as previously described [40,43].

### 3.2. Synchrotron Radiation Circular Dichroism

The protein sample was prepared by dissolving 0.4 mg/mL (0.019 µM) of VVTL1 in a model wine solution (MWS) that was composed by 12% (*v*/*v*) ethanol in a meso-tartaric acid solution (5 g/L) adjusted to pH 3.2 with HCl 1M. Polyphenol stock solutions were obtained dissolving an appropriate amount of each compound in MWS (final concentration 0.038 µM). The molarity of the WTE was calculated while considering the average mass of the phenolic compounds present in the extract, as measured by UHPLC-Q/TOF analysis, as previously described [59].

The Synchrotron Radiation Circular Dichroism (SRCD) spectra in the far-UV region were collected at the Diamond B23 beamline module end station B (Diamond Light Source Ltd., Didcot, Oxfordshire, UK) using an integration time of 1 s, 1 nm digital resolution, and 39 nm/min. scan speed. Different monochromator slit widths (0.5–1.0 mm) were used according to the experiment. The spectra were recorded using a 0.02 cm path length Suprasil cell (Hellma Analytics, Müllheim, Germany).

Thermal stability was monitored recording the far-UV SRCD spectrum in the 5–70 °C temperature range, at 5 °C increments with 5 min. equilibration time, while using a Quantum Peltier temperature controller.

Protein UV photo-denaturation assay was performed recording twenty consecutive repeated scans for each sample at 20 °C. SRCD spectra were processed and analyzed using the CDApps software [45].

The near-UV spectra were recorded using a nitrogen flushed Jasco J-715 spectropolarimeter (Tokyo, Japan) using a 1.0 cm quartz cells (Hellma Analytics, Müllheim, Germany). The calculated near-UV CD spectra were obtained by adding the near-UV CD spectrum of the VVTL1 protein, subtracted of the baseline contribution, to the near-UV CD spectrum of the corresponding polyphenol, also being corrected for the baseline contribution.

## 4. Conclusions

Despite Circular Dichroism being commonly used to study protein binding to ligands [60], this technique was never applied for the study of the interactions of wine proteins with polyphenols, even if this has great relevance for its effects on wine quality. In this study, Synchrotron Radiation Circular Dichroism (SRCD) was applied for the first time using a model system, in which the major wine protein (VVTL1) was analyzed in absence or presence of selected phenolic compounds as well as of a total tannins extract directly obtained from a white wine. SRCD measurements were executed on samples that were subjected to both thermal and UV denaturation, in order to increase the quantity of information obtainable by this spectroscopic technique.

The ratio between the amounts of proteins and phenolics modulates the possibility to have cross-linking between these components, leading to the formation of insoluble complexes, according to the commonly accepted model for protein/polyphenols interactions [61]. Therefore, in this study, a protein/phenolics ratio that was much higher than that found in real wines was intentionally used to prevent potential cross-linking phenomena that would have hindered the accurate analysis of the protein structure, as affected by the presence of polyphenols, which was the aim of this investigation.

The hypothesis that VVTL1 can interact with different phenolic compounds was proven. VVTL1 is a protein characterized by a high hydrophilicity and a globular shape [24], characteristics that generally make the binding of a protein with polyphenols unfavorable. However, our CD findings indicate that VVTL1 can interact with a variety of phenolic compounds that are generally present in wines. Additionally, it was highlighted that these interactions result in strong modifications of the environment of the aromatic residues of the VVTL1, while the secondary structure did not show major changes as compared to the protein alone, a fact that is attributable to the rigid structure peculiar of this class of proteins [24,41]. In particular, these interactions were proven to occur at temperatures that are commonly used in winemaking, which suggests that the presence of polyphenols can affect the structure of proteins in real wines. Consequently, these modifications should be involved in all of the phenomena in which wine proteins play a key role, including protein stability [12,62], modulation of wine organoleptic properties [43], and sparkling wine foamability [63].

Additionally, the interaction with polyphenols also affected the thermal behavior of the VVTL1, which has been indicated as a major factor that is involved in the mechanism of protein stability in wines [12]. If the interactions of proteins with polyphenols affect protein thermal stability, the quantity and nature of the phenolics should be considered when studying the stability of different wines. Indeed, each wine has its peculiar phenolic profile, and this is likely to affect the level of stability/instability of its proteins. For example, in white wines, the heat denaturation of proteins is considered to be the culprit of haze formation, a phenomenon that generally does not occur in red wines that naturally contain larger quantities of phenolics of different types.

The results presented here can be useful to study the relationship existing between wine protein content, composition, and stability. Indeed, it is likely that the phenolic composition affects the behavior of the same proteins in different wines, thus explaining why the protein content does not fully correlate with the wine hazing potential [64].

Therefore, future work in this field should take phenolic composition into great account. Obviously, given that wine instability is due to different isoforms of thaumatin-like proteins and chitinases, more work is needed to elucidate the impact of the different classes of wine phenolics on the stability of each of these proteins.

## Figures and Tables

**Table 1:** Secondary structure content of VVTL1 (0.400 mg/mL) alone or in presence of polyphenols at 5, 20 and 70 °C. The secondary structure content was calculated by CDApps [45] using CONTINLL algorithm [46]. Unord.: unordered structure.

VVTL1	% Secondary Structure Content											
	5 °C				20 °C				70 °C			
	α-helix	β-sheet	turns	Unord.	α-helix	β-sheet	turns	Unord.	α-helix	β-sheet	turns	Unord.
**Alone**	2	44	22	32	3	45	22	29	6	30	19	49
**+ PB1**	0	44	21	30	0	37	22	38	0	18	12	64
**+ PB2**	4	41	22	32	4	41	22	33	2	15	14	53
**+ Q**	5	44	22	29	5	43	22	30	5	33	16	46
**+ R**	5	43	22	30	4	44	22	30	4	34	16	46
**+ WTE**	4	41	22	33	4	43	22	31	7	20	12	60
**+ TA**	4	46	23	27	5	44	22	29	7	24	14	55

**Table 2:** Melting temperatures (T_M_) values (°C) of VVTL1 alone and in presence of phenolic compounds. T_M_ data were calculated using the software OriginPro 2018 (OriginLab Corporation), starting from the melting curves reported in Figure S2.

Sample	T_M_ 1	T_M_ 2
**VVTL1 alone**	32.2	53.9
**VVTL1 + PB1**	42.8	58.5
**VVTL1 + PB2**	19.5	55.4
**VVTL1 + Q**	18.8	47.0
**VVTL1 + R**	12.4	48.0
**VVTL1 + WTE**	11.2	58.3
**VVTL1 + TA**	29.3	53.3

**Table 3:** Secondary structure content of VVTL1 (0.400 mg/mL) alone or in the presence of phenolic compounds after 1 and 20 scans in UV photo-denaturation assay. Secondary structure was calculated by CDApps [45] using CONTINLL algorithm [46].

VVTL1	% Secondary Structure							
	After 1 Scan				After 20 Scans			
	α-Helix	β-Sheet	Turns	Unordered	α-Helix	β-Sheet	Turns	Unordered
**alone**	6	41	22	31	6	32	17	45
**+ PB1**	5	43	24	28	7	25	24	44
**+ PB2**	4	43	22	31	9	38	23	30
**+ Q**	5	43	22	30	6	39	20	35
**+ R**	5	43	22	30	6	41	20	33
**+ WTE**	4	43	23	31	17	21	19	44
**+ TA**	4	46	23	27	4	38	19	38

## Supplementary Materials

The following are available online: Figure S1: Plot of secondary structure content of VVTL1 alone or in presence of 2 eq. of polyphenols (indicated) versus temperature. Secondary structure content was determined from SRCD data by CDApps [45] using CONTINLL algorithm [46]. VVTL1 concentration was 0.400 mg/mL. Figure S2: Melting curves of VVTL1 alone or in presence of 2 eq. of polyphenols (indicated). Curves were obtained plotting the ΔSRCD signal at 195 nm of VVTL1 versus temperature. Figure S3: Far-UV SRCD spectra (20 repeated scans collected at 20 °C) of VVTL1 alone or in presence of different polyphenols. SRCD spectra were recorded using a Suprasil 0.02 cm cell (Hellma) filled with 60 µL of solution, integration time 1 s, 1 nm digital resolution, 39 nm/min scan speed and monochromator slit widths to 1.2 nm bandwidth. Protein concentration 0.400 mg/mL in MWS. Figure S4: Changes in the SRCD signal at 195 nm versus the number of scans of VVTL1 alone and in presence of polyphenols (indicated).
