# Supplementary material for: The Secondary Structure of a Major Wine Protein is Modified upon Interaction with Polyphenols
Source: Molecules. 2020 Apr 3;25(7):1646. doi: 10.3390/molecules25071646 (PMC7180857; doi:10.3390/molecules25071646)
Supplement: Supplementary file 1 [file molecules-25-01646-s001.pdf]

Supplementary Materials

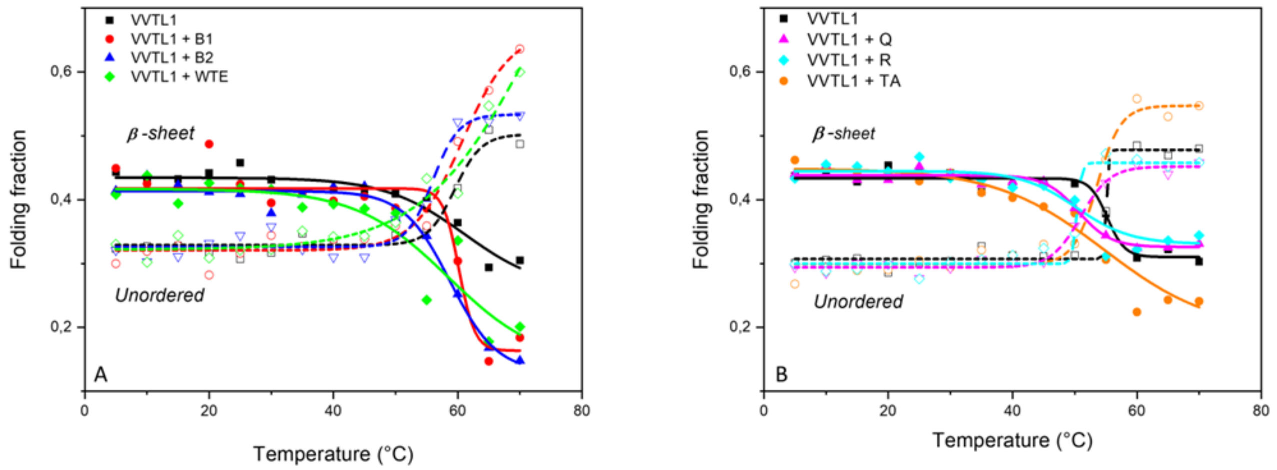

Figure S1. Plot of secondary structure content of VVTL1 alone or in presence of 2 eq. of polyphenols (indicated) versus temperature. Secondary structure content was determined from SRCD data by CDApps [45] using CONTINLL algorithm [46]. VVTL1 concentration was 0.400 mg/mL

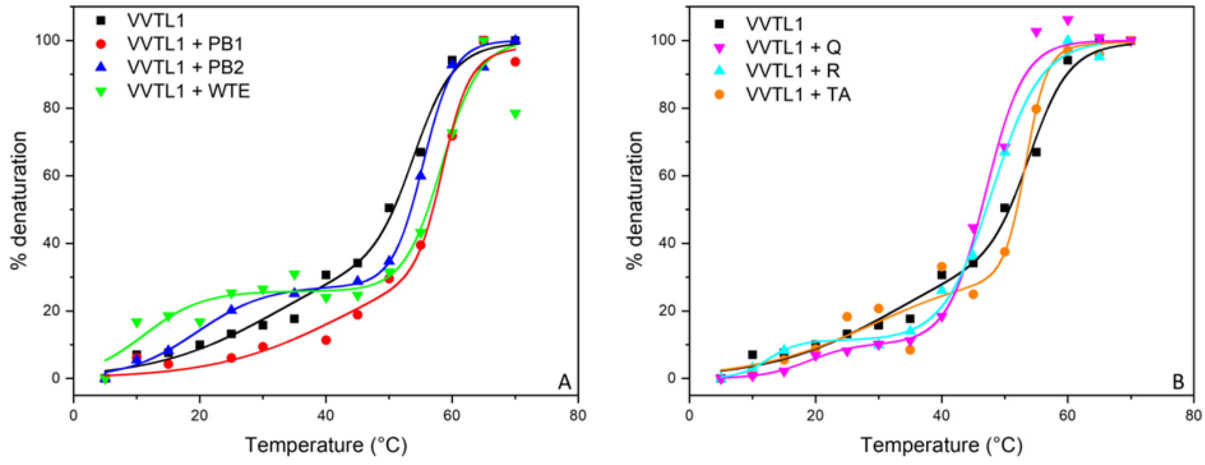

Figure S2. Melting curves of VVTL1 alone or in presence of 2 eq. of polyphenols (indicated). Curves were obtained plotting the ΔSRCD signal at 195 nm of VVTL1 versus temperature.

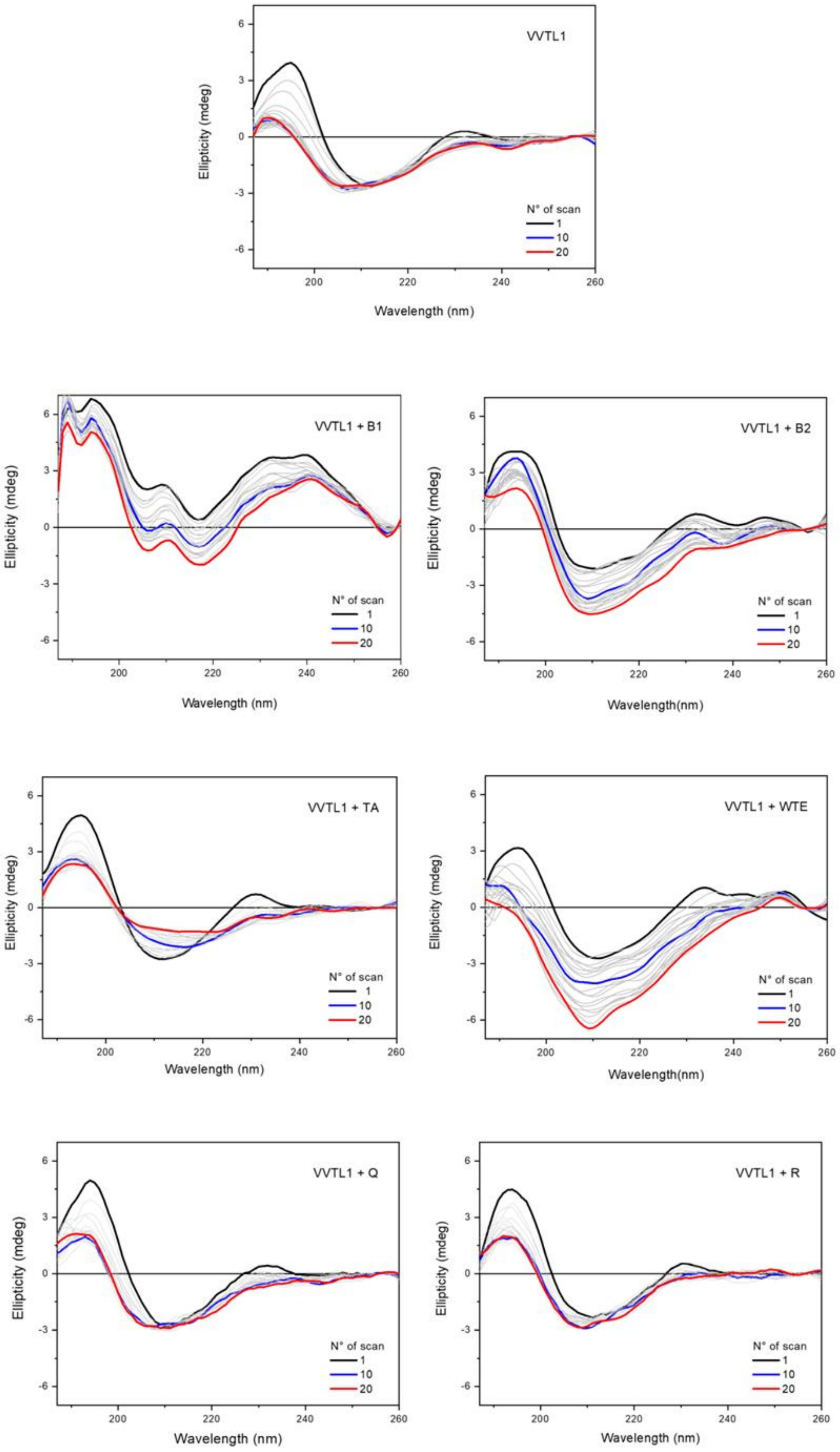

Figure S3. Far-UV SRCD spectra (20 repeated scans collected at 20°C) of VVTL1 alone or in presence of different polyphenols. SRCD spectra were recorded using a Suprasil 0.02 cm cell (Hellma) filled with 60 μL of solution, integration time 1 s, 1 nm digital resolution, 39 nm/min scan speed and monochromator slit widths to 1.2 nm bandwidth. Protein concentration 0.400 mg/mL in MWS.

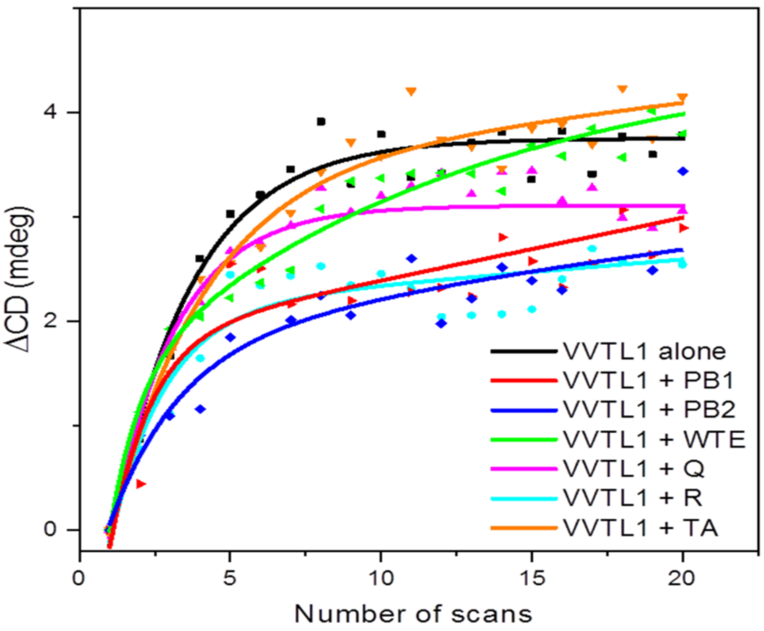

Figure S4. Changes in the SRCD signal at 195 nm versus the number of scans of VVTL1 alone and in presence of polyphenols (indicated).
